# Supplementary material for: Causal interplay between lactose intolerance and gut microbiota: a combined bidirectional Mendelian randomization and in vivo validation study
Source: Front Nutr. 2026 Jun 1;13:1803337. doi: 10.3389/fnut.2026.1803337 (PMC13265576; doi:10.3389/fnut.2026.1803337)
Supplement: Supplementary file 1 [file Data_Sheet_1.zip › supplementary materials/Forward/forest plot/ebi-a-GCST90027834.finngen_R12_E4_LACTONAS.pdf]

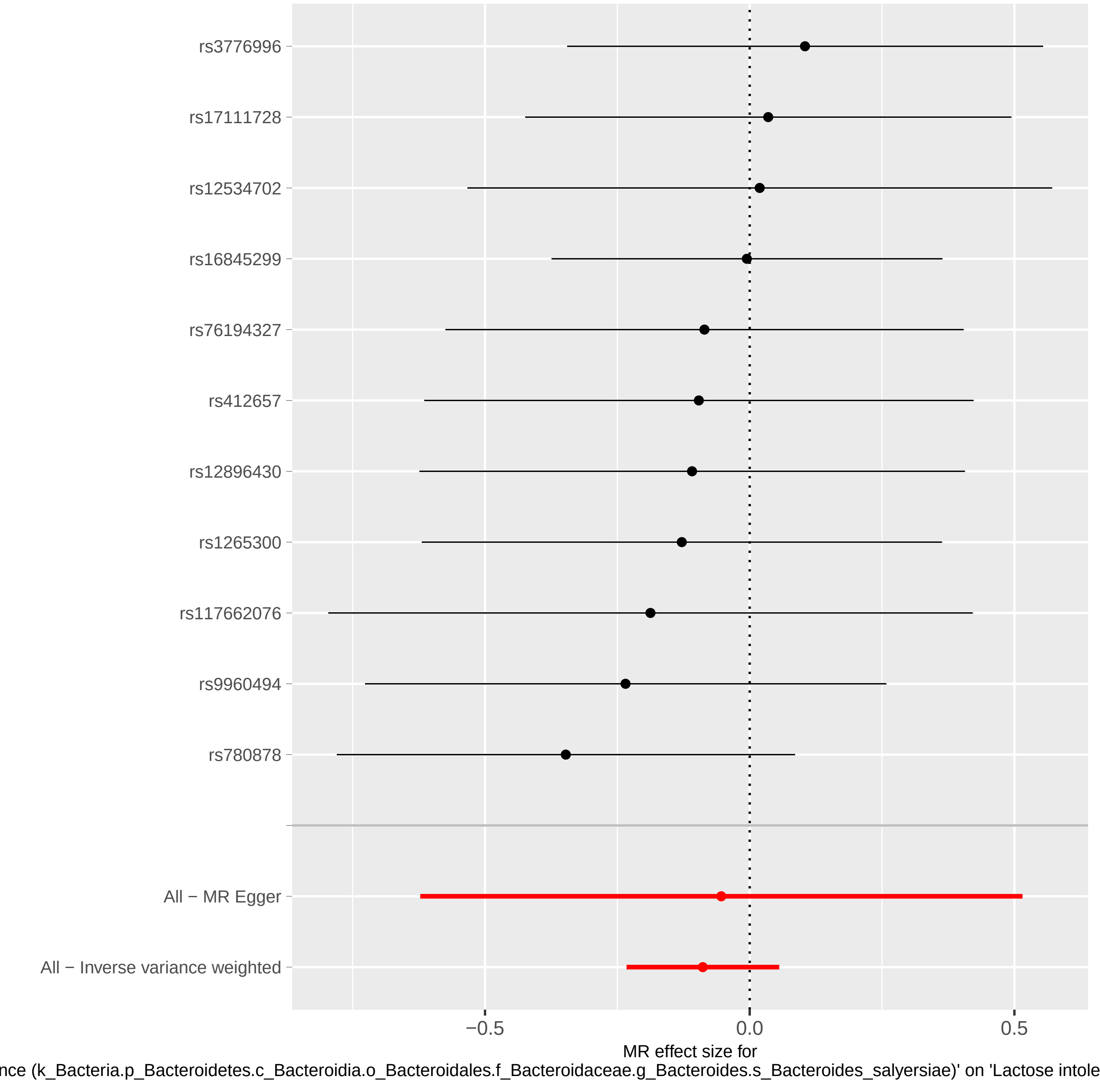
rs3776996
rs17111728
rs12534702
rs16845299
rs76194327
rs412657
rs12896430
rs1265300
rs117662076
rs9960494
rs780878
All − MR Egger
All − Inverse variance weighted
−0.5
0.0
0.5
MR effect size for
nce (k_Bacteria.p_Bacteroidetes.c_Bacteroidia.o_Bacteroidales.f_Bacteroidaceae.g_Bacteroides.s_Bacteroides_salyersiae)' on 'Lactose intole
